# ICNP® terminological subset for people with diabetic foot ulcer in primary health care

**DOI:** 10.1590/0034-7167-2022-0668

**Published:** 2023-11-27

**Authors:** Halene Cristina Dias de Armada e Silva, Sonia Acioli, Patricia dos Santos Claro Fuly, Silvia Maria de Sá Basilio Lins, Juliana Otaciana dos Santos, Danielle Lemos Querido, Alessandra Conceição Leite Funchal Camacho, Harlon França de Menezes

**Affiliations:** I Governo do Estado do Rio de Janeiro, Secretaria de Estado de Saúde. Rio de Janeiro, Rio de Janeiro, Brazil; II Universidade do Estado do Rio de Janeiro. Rio de Janeiro, Rio de Janeiro, Brazil; III Universidade Federal Fluminense. Niterói, Rio de Janeiro, Brazil; IV Universidade Federal do Rio de Janeiro. Rio de Janeiro, Rio de Janeiro, Brazil

**Keywords:** Nursing Process, Standardized Nursing Terminology, Diabetes *Mellitus*, Diabetic Foot, Primary Health Care

## Abstract

**Objectives:**

to elaborate an ICNP® terminological subset for people with diabetic foot ulcers in Primary Health Care.

**Methods:**

this is a methodological study that followed five steps: 1) Identification of relevant terms for the patients through an integrative literature review and official documents in the area; 2) Mapping of terms identified with ICNP® terms; 3) Construction of statements of nursing diagnoses, outcomes and interventions; 4) Structuring of a terminological subset with the Self-Care Deficit Theory; and 5) Content validity of statements constructed with nurses from a programmatic area in Rio de Janeiro.

**Results:**

the subset developed is composed of 81 diagnoses/outcomes and 583 nursing interventions, organized into universal, change and development requirements.

**Conclusions:**

the subset on screen was predominantly composed of statements inserted in self-care requirements related to health changes, reinforcing the importance of quality of life and recovery.

## INTRODUCTION

Diabetes *Mellitus* (DM) is a metabolic disease and makes up the group of Chronic Noncommunicable Diseases (NCDs), which are responsible for 71% of deaths worldwide^(1)^. In Brazil, NCDs are equally relevant, being the fifth country with the highest incidence of diabetes in the world, with 16.8 million adult patients (20 to 79 years old). The estimated incidence of the disease in 2030 reaches 21.5 million^(2)^.

Among its various chronic complications, ulceration and amputation of extremities, arising from the worsening of diabetic foot, are some of the most serious and have the greatest socioeconomic impact, and are, unfortunately, still frequent in our population^(3)^. Diabetic foot is defined as the presence of infection, ulceration and or destruction of deep tissues associated with neurological abnormalities and various degrees of peripheral vascular disease in people with DM^(4)^.

Given the scenario presented, and understanding that nurses make up the multidisciplinary team in Primary Health Care (PHC), there is an urgent need to implement care that considers, in addition to the disease and the individual, their family, environmental, social, economic context and cultural^(5)^. In this way, it is observed that people even registered in family health teams develop ulcers, with amputation as a complication, and it is possible to reflect on possible weaknesses in nursing care systematization interrelated with the essential attributes of PHC such as access, coordination of care, longitudinality and comprehensiveness of actions.

In this regard, self-care is one of the most relevant aspects in the treatment of people with diabetes, given that changing behavior improves health status and reduces the chances of complications, such as the presence of ulcers. Furthermore, self-care has been seen as a trend in studies for people with diabetes^(6-7)^. Therefore, the adoption of Dorothea Orem’s Self-Care Deficit Theory (SCDT) and the concepts of universal self-care requirements, of development and of health deviation belong to the study, since this framework has as assumptions that people have the potential to develop intellectual and practical skills, in addition to the essential motivation for self-care through responsibility for health^(8)^.

Thus, it is imperative that nurses use standardized terminology, such as the International Classification for Nursing Practice (ICNP^®^), which allows them to name, classify and link phenomena that describe the essential elements of professional practice, what are the judgments about certain human and social needs (nursing diagnoses), and what nursing does to positively influence such diagnoses (nursing actions/interventions), to produce results sensitive to interventions (nursing outcomes)^(9)^.

It is noteworthy that, even with an ICNP^®^ subset for people with diabetes in specialized care, it differs from the current one, as it adopted another theoretical framework, covered the general repercussions of health condition and used a previous version of ICNP^®(10-11)^. With this, the importance of current subset is reinforced for updating nurses’ practice as well as for ICNP^®^ evolution, since it proves to be innovative, thus being able to provide an expansion for the specialized area of study.

ICNP^®^ use can be strengthened through the construction of terminological subsets consisting of nursing diagnosis, outcome and intervention statements for a group of patients and selected health priorities^(12-13)^, as, in this study, people with diabetic foot ulcers in PHC, its development being indeclinable, since so far there is no ICNP^®^ subset for this population and health priority.

## OBJECTIVES

To elaborate an ICNP^®^ terminological subset for people with diabetic foot ulcers in PHC.

## METHOD

### Ethical aspects

The study was conducted in accordance with national and international ethics guidelines, and was approved by the Research Ethics Committee of the *Universidade do Estado do Rio de Janeiro*. The Informed Consent Form was obtained from all individuals involved in the study in writing.

### Study design, period, and place

This is a methodological study, carried out between August 2019 and December 2020, which followed the Brazilian method guidelines for developing terminological subsets^(14)^: 1) identification of relevant terms for patients through an integrative literature review and search in official documents in the area; 2) mapping of identified terms with ICNP^®^ terms, version 2019/2020^(9)^; 3) construction of nursing diagnosis, outcome and intervention statements; 4) structuring of an ICNP^®^ terminological subset for people with diabetic foot ulcers with SCDT^(8)^; and 5) content validity of statements constructed with PHC nurses from a programmatic area in Rio de Janeiro.

### Population and elegibility criteria

For the content validity stage of the statements present in the subset, we searched for expert nurses working in 35 PHC units of a programmatic area (PA) in the city of Rio de Janeiro as well as those working in the health coordination of the referred PA. Nurses were selected through a search carried out in the Brazilian National Register of Health Establishments.

Nurses with a minimum of two years of experience in PHC, working in management or assistance were included, being those linked to the diabetes program of their units, and knowledge about language systems/nursing diagnoses. Nurses who were on sick leave were excluded.

For sample calculation, a confidence level of 95% was considered, with a sampling error of 15%, resulting in 21 experts^(15)^. Nurses who did not complete the entire instrument or who did not respond to the e-mail within the pre-established period were considered as research dropouts.

### Study protocol

In the first stage, the identification of terms was carried out by the main researcher, performing an integrative literature review that included official documents in the area. Therefore, a review was carried out in the Medical Literature Analysis and Retrieval System Online (MEDLINE), Latin American and Caribbean Literature on Health Sciences Information (LILACS) and Cumulative Index to Nursing and Allied Health Literature (CINAHL) databases, using of different combinations of health descriptors “Diabetic Foot” and “Nursing”, using Boolean operators.

With regard to official documents, these documents were chosen because they are reference guides to multidisciplinary health teams for the care of people with diabetes and/or diabetic foot in the different world care network scenarios. National and international publications in the form of catalogues, manuals or documents guiding care for people with diabetes and/or diabetic foot, published in the last ten years were included. Publications that, despite the focus on diabetes and/or diabetic foot, were in the format of a booklet to be completed by the user, publications on epidemiological data, publications aimed at community health agents and material aimed at managers, were excluded.

The selected publications underwent an adaptation process with removal of sections with low potential for relevant terms, such as titles, authors, acknowledgments, abstracts, methodology, references, footnotes and information about the authors as well as official documents, being removed of these, there were also other complications of diabetes that did not refer to the diabetic foot.

The articles and documents that were in other languages were translated in full into Portuguese, by a proficient translator, for later unification with the articles and documents in the Portuguese language, fulfilling the grouping of publications in a single Word^®^ file with subsequent conversion to the portable document format PDF (Portable Document Format), constituting the corpus of the study at this time of the research.

The terms were extracted using a computational tool called PorOnto^(16)^, which processed a list in Excel^®^ of terms according to their frequency of appearance. The terms were arranged in alphabetical order for better visualization, with subsequent normalization and standardization of inflections of gender, number and degree, in order to identify and remove repetitions of terms, being done by manual sorting.

With these terms in hand, the second stage was performed, where the normalized terms were cross-mapped with ICNP^®^ and its current version, 2019/2020. Manual mapping of terms/concepts found in the literature was carried out, with the primitive terms/concepts of ICNP^®^ Seven-Axis Model, paying attention to their definitions, in order to compare them and establish semantic equivalence and exclusion of synonyms. In cases of semantic doubts, a Portuguese language dictionary was used, in comparison to the definitions contained in ICNP^®^, in order to reduce difficulties and/or occurrence of errors in the interpretation.

It became necessary to use the International Organization for Standardization (ISO) 12300:2016, which addresses the standards for mapping between terminological systems, providing subsidies for the creation of clinical terminologies or subsets of specific use^(17)^. The result of mapping generated a new Excel^®^ spreadsheet with primitive concepts with similarity/similarity and coverage in the ICNP^®^ 2019/2020 version.

As for the third stage, the construction of nursing diagnosis/outcome and intervention statements was carried out, using the following evidence: list of specialized language terms; ICNP^®^ Seven-Axis Model, version 2019/2020; ISO 18.104:2014 standard; the SCDT; ICNP^®^ list of nursing diagnoses/outcomes and interventions, version 2019/2020. It should be noted that, for the construction of diagnosis/outcome statements, a term from the Focus axis and a term from the Judgment axis were included with a single descriptor that was equivalent to focus and judgment or just a clinical finding that could represent an altered state, function altered or even modification in behavior. To elaborate the operational definitions of constructed statements, three stages were covered: literature review; concept meaning mapping; and operational definition affirmation^(18)^. The codes of the diagnostic statements that were constant in ICNP^®^ were presented.

For the elaboration of statements of nursing interventions, a term from the Action axis and target terms were used. Moreover, suggested nursing interventions based on participating nurses’ experience were included.

The fourth stage involved structuring the statements and their allocation, according to SCDT requirements, as follows: diagnoses classified according to the universal requirements of self-care; developmental self-care requirements; and self-care requirements related to health changes^(8)^.

Nursing intervention statements were classified according to the following nursing systems: fully compensatory system (when the person is unable to engage in self-care actions, and nurses are the main contributor so that all self-care requirements are met satisfied); partially compensatory system (when nurses and people develop self-care methods); and support-education system (when people have the skills to perform and/or learn to perform self-care-oriented methods, with nurses’ role being primarily consultative).

Finally, in the fifth stage, there was content validity by expert nurses. The distribution of the questionnaires took place through the preparation of a notebook for experts in May 2020, by email, with guidance on completing it. This included an invitation letter to participate in the study, the Informed Consent Form (ICF), in two copies, experts’ characterization instrument and the data collection instrument.

The collection instrument consisted of nursing diagnoses/outcomes and interventions, and the expert nurses agreed by marking an “x” on a Likert-type scale, as described below. Furthermore, they filled in suggestions for writing statements regarding their use in clinical practice. This instrument was created based on methodological research that carried out terminological subsets, with no need for validity. The turnaround time was 30 days.

### Data analysis and treatment

Data were analyzed using descriptive statistics. In order to analyze the degree of agreement among experts, the Content Validity Index (CVI) was used, creating a five-point Likert scale (1 = not at all relevant; 2 = slightly relevant; 3 = very relevant; 4 = relevant; 5 = very relevant) to measure the relevance of statements for nursing practice applied to people with diabetic foot ulcers. Then, a weighted arithmetic mean of the scores assigned by each specialist was calculated, in order to obtain the CVI. In this regard, diagnoses with a CVI ≥ 0.80 were considered validated.

## RESULTS

The first stage of the study resulted in a sample composed of 62 articles. Five official documents were also used, two from the Brazilian Ministry of Health, one from the Brazilian Society of Diabetes, one from the Portuguese Ministry of Health and one from the Peruvian Ministry of Health.

Regarding expert nurses, there was a prevalence of women (81%), aged between 30 and 50 years (86%), with professional practice time between 6 and 10 years, and equally represented with 38% aged 16 years or more. Among nurses who worked in care and management, there was a practically equal average, with care nurses represented with 52% in the study sample. Most nurses have only specialization (81%).

The extraction of terms found in the productions for people with diabetic foot ulcer resulted in 12,696 terms, which were excluded from repetitions, normalized and standardized in relation to ICNP^®^. At the end of this procedure, 392 related terms remained, of which 305 were nouns, 39 were adjectives and 48 were verbs.

With these data in hand, a list of 98 nursing diagnosis/outcome (ND/NO) statements was constructed for people with diabetic foot ulcers in PHC, which underwent similarity analysis, with repetitions being removed according to the definition of them. Thus, 81 statements were maintained, of which five (6%) were positive, nine (11%) risk and 67 (83%) negative. Of the 81 ND/NO statements constructed, 58 (71%) are constant in ICNP^®^ as combined diagnoses, present in the Focus axis, or similar to the constant statements, and 23 (29%) are not included in the classification.

Among the 23 statements not included in ICNP^®^, 17 (21%) were classified as more restricted and six (7%) without agreement in relation to ND/NO or Focus axis terms. No statements classified as more comprehensive were found. All diagnoses were validated by experts, considering a CVI ≥ 0.8 in the general mean.

A relevant data evidenced was that, among the six statements that did not present validity with the ICNP® statements, five were classified by nurses with CVI = 0.9, which are “Blister”, “Callus”, “Absent Hair Growth”, “Maceration” and “Increased Interdigital Humidity”, and one with CVI = 0.8, being “Altered Skin Color”.

The diagnoses were classified according to Orem’s self-care requirements, and are presented in Chart 1, where: 7% of constructed diagnoses correspond to universal self-care requirements; 51%, self-care requirements related to health changes; and 42%, self-care requirements related to the development.

**Chart 1:** Distribution of nursing diagnoses/outcomes for people with diabetic foot ulcers in primary care according to self-care requirements, Rio de Janeiro, Rio de Janeiro, Brazil, 2022

Universal self-care requirements
Impaired Self Feeding (10000973); Positive Family Support (10045702); Impaired Capacity for Socialization; Impaired Cognition (10022321); Effective Social Support (10045794); Impaired Sexual Performance (10001288).
**Self-care requirements related to health changes**
Blister; Callus; Impaired Wound Healing; Altered Skin Color; Effective Pain Management; Absent Hair Growth; Pain (10023130); Peripheral Oedema (10027482); Impaired Balance (10047170); Rash (10016388); Fatigue (10000695); Weakness (10022880); Low Pedal Pulse Rate; Impaired Peripheral Neurovascular Function (10023153); Hematoma (10008931); Hyperglycaemia (10027550); Hyperthermia (10000757); Hypoglycaemia (10027566); Infection (10023032); Inflammation (10029927); Impaired Skin Integrity (10001290); Maceration (10011493); Impaired Gait (10001046); Impaired Metabolism; Dry Skin (10047073); Impaired Peripheral Tissue Perfusion (10044239); Altered Blood Pressure (10022954); Severe Plantar Pressure; Impaired Musculoskeletal System Process (10012773); Risk for Chills; Absent Pedial Pulse Rate Risk; Risk for Hyperthermia (10027328); Risk for Fall (10015122); Risk for Tachycardia; Ulcer Bleeding; Stress Overload (10021742); Overweight (10027300); Impaired Sleep (10027226); Impaired Diet Tolerance; Diabetic Ulcer (10042181); Impaired Vision (10022748).
**Developmental self-care requirements**
Impaired Acceptance of Health Status (10029480); Impaired Adaptation (10022027); Alcohol Abuse (10022234); Anxiety (10000477); Positive Care Attitude (10022275); Negative Self Image (10022724); Low Self Control (10027469); Impaired Community Capacity to Manage Regime (10000892); Ability to Perform Impaired Hygiene (10000987); Impaired Ability to Perform Leisure Activity (10040351); Impaired Ability to Participate in Care Planning (10035134); Aggressive Behavior; Impaired Communication (10023370); Risk Housing Condition; Impaired Health Knowledge; Lack of Knowledge of Diagnostic Test (10021987); Conflicting Cultural Belief (10022397); Conflicting Religious Belief (10021757); Self Care Deficit (10023410); Unrealistic Expectation about Treatment (10042357); Fear (10000703); Non Adherence to Exercise Regimen (10022657); Non Adherence to Therapeutic Regimen (10022155); High Care Need; Emotional Problem (10029839); Impaired Nail Care Regimen; Inadequate Income (10022563); Low Treatment Responsiveness; Risk for Self Destructive Behaviour (10015302); Risk for Negative Quality of Life (10040945); Risk for Suicide (10015356); Suffering (10025588); Tobacco Abuse (10022247); Increased Interdigital Humidity.

For each ND/NO statement, NI statements were constructed, also using the essential and derived attributes of PHC, totaling 583 NI statements in this study. It is noteworthy that, among the 583 statements, some appeared according to relevance related to more than one ND/NO.

Considering that in this study 81 diagnoses and 585 nursing interventions were constructed, those that presented CVI = 1 were selected for presentation in Chart 2, and in Chart 3, the diagnoses that did not have agreement with ICNP^®^ diagnoses, as they were considered most relevant by the authors.

**Chart 2:** Examples of statements of diagnoses/outcomes contained in the International Classification for Nursing Practice and nursing interventions for people with diabetic foot ulcers in Primary Health Care provided by Orem’s nursing systems, Rio de Janeiro, Rio de Janeiro, Brazil, 2022

Nursing diagnosis/outcome	Nursing interventions
Self-care requirement related to health changes	
Hypoglycemia	**Fully Compensatory System:** 1. Carry out glycemic test in consultations; 2. Assess patients’ knowledge about the therapeutic scheme of the drugs in use and the duration of action of each medication; **Support System - Education:** 3. Encourage patients to maintain glycemic control within normal standards; 4. Encourage self-monitoring of blood glucose levels; 5. Teach patients how to proceed in case of low blood glucose values; 6. Clarify symptoms of hypoglycemia and investigate individual symptoms; 7. Guide patients to seek a health unit in case of history of recurrent hypoglycemia to identify the causes and adjust the therapeutic regimen. 8. Guide patients to check capillary blood glucose before performing important daily tasks, if insulin dependent; 9. Warn patients about the importance of blood glucose monitoring before, during and after physical activity.
Overweight	**Fully Compensatory System:** 1. Monitor weight; 2. Interconsultation with a nutritionist; **Partially Compensatory System:** 3. Obtain data on food and fluid intake behavior; 4. Establish with patients a food plan suited to their lifestyle; **Support System - Education:** 5. Encourage food intake according to nutritional needs and food preferences, respecting the nutritionist’s prescription; 6. Clarify the negative consequences of excessive food intake to patients; 7. Guide patients to control weight; 8. Discuss with patients about the importance of adhering to healthy eating; 9. Teach patients how to select food away from home; 10. Assess the causes of impaired nutritional intake; 11. Assess the need to change eating habits; 12. Advise patients on the importance of maintaining the proper weight; 13. Clarify possible complications in case of overweight; 14. Assess possible anxiety disorder.
**Developmental self-care requirement**	
Impaired Nail Care Regimen	**Fully Compensatory System:** 1. Prescribe nail care regimen; 2. Assess nail trimming; 3. Assess the presence of ingrown toenails; 4. Assess the need for correction of ingrown toenails; **Support System - Education:** 5. Instruct patients to cut the nails straight and not too close, preferably after bathing with round-tipped scissors; 6. Guide patients not to scratch the skin; 7. Instruct patients not to try to correct the ingrown toenails on their own.

**Chart 3:** Examples of statements of diagnoses/outcomes not contained in the International Classification for Nursing Practice and nursing interventions for people with diabetic foot ulcers in Primary Health Care provided by nursing systems by Orem, Rio de Janeiro, Rio de Janeiro, Brazil, 2022

Nursing diagnosis/outcome	Nursing interventions
Self-care requirement related to health changes	
Blister	**Fully Compensatory System:** 1. Investigate the presence of blisters at every appointment; **Support System - Education:** 2. Guide the patients to self-examination in search of blisters; 3. Guide prevention of blisters; 4. Advise not to pop blisters.
Callus	**Fully Compensatory System:** 1. Investigate the presence of calluses at every appointment; 2. Assess the need for callus removal; 3. Forward to the occupational therapist to make suitable shoes; **Support System - Education:** 4. Encourage patients to perform self-examination in search of calluses; 5. Explain to patients not to use chemical agents or plasters to remove calluses; 6. Advise patients not to remove calluses.
Absent Hair Growth	**Fully Compensatory System:** 1. Observe hair distribution; 2. Correlate the absence of hair with other symptoms that indicate reduced blood perfusion; 3. Monitor hair loss or absence; **Support System - Education:** 4. Explain causes of reduced hairiness; 5. Stimulate adherence to treatment to improve blood perfusion.
Maceration	**Fully Compensatory System:** 1. Avoid excessive skin moisture, paying attention to the use of solutions and dressings only in the area of the lesion; 2. Do not perform debridement in the macerated area; 3. Pay attention to the removal of the adhesive tape when changing dressings so as not to increase the lesion; **Support System - Education:** 4. Guide patients and family regarding the correct performance of the dressing.
Altered Skin Color	**Fully Compensatory System:** 1. Investigate causes for altered skin color such as peripheral artery disease; **Support System - Education:** 2. Teach patients and family members in groups, consultations or home visits to monitor skin with signs of cyanosis, redness and shine.
**Developmental self-care requirement**	
Increased Interdigital Wetness	**Fully Compensatory System:** 1. Perform inspection of patients’ feet at each appointment; 2. Investigate the presence of interdigital moisture; **Support System - Education:** 3. Guide the drying of the feet as well as the interdigital spaces after bathing; 4. Inform patients not to use moisturizer in the interdigital spaces.

## DISCUSSION

The literature points out that the elaboration of a terminological subset establishes a systematic practice, since the affected needs of individuals can be addressed, assessed and encouraged to resolve^(19-20)^. Thus, the development of a subset for people with diabetic foot ulcers in PHC favors nurses’ decision-making at the first individual and collective point of contact, offering promotion, prevention, treatment, rehabilitation and maintenance. Thus, the subset encompasses its own language based on the daily experiences of nurses’ work process, contributing to a usual and homogeneous language of their assessment.

The first stages of the study revealed that the terminology, although not deeply complied with in clinical practice, is glimpsed in the literature and official documents, representing everyday situations. Some terms were classified as non-constant, such as “diabetic foot”, “neuropathy”, “deformity”, “sensitivity”, which reflect progress. Through the contribution of studies and research, they have been fundamental for standardizing and expanding the standardized language for nursing practice^(21)^.

Among the sets of statements related to health changes, there are the diagnoses “Hypoglycemia” and “Overweight” contained in ICNP^®^ and validated for this subset. These statements represent a common reality in PHC, as people can have their glycemic and weight control impaired by numerous difficulties. As can be seen, the attitude towards the disease is essential in the adoption and maintenance of certain patterns of behavior, as it represents a requirement for the adoption of self-care actions, contributing to reduction of stress associated with the disease, greater clarity of treatment, improvement in quality of life and a sense of self-efficacy and positivity in relation to health^(22)^.

Therefore, it is important for nurses to consider, in their assessment, the adoption of measures for self-care in the health of people living with diabetes and its complications. Self-care is seen as one of the main components of treatment that people must take up, requiring that they have the knowledge and skills to develop actions that are essential for maintaining the quality of metabolic control^(23)^.

A study carried out in southern Brazil that investigated the factors associated with glycemic control in people with DM revealed that individuals aged between 50 and 69 years, those using insulin, the obese and those at risk for foot ulceration showed higher prevalence rates of alteration in glycated hemoglobin^(24)^. That said, the relevance of nurses’ work in PHC is understood, with a view to supporting measures for self-care as well as for a correct assessment, adoption of conducts for effective treatment and health education, aiming at adequate care in prevention and treatment from the ulcer.

Other statements of nursing diagnoses that achieved a good validity rate and are not included in ICNP^®^ were “Blister” and “Callus”. These diagnoses converge with the Brazilian study that points out that calluses and calluses, which are considered pre-ulcerative lesions, are classified as predictive of ulcerations. These injuries can occur due to musculoskeletal alterations that, associated with insensitive feet, constitute an important pathway for ulceration^(25)^.

With these indications in hand, it is important for nurses to detect any dermatological changes, such as the presence of skin thickening (keratosis), fissures, dry skin, blisters, active ulcers, nail changes, maceration and interdigital fissures^(25)^. These represent diagnoses also addressed in the subset and reinforce the importance of instituting interventions that cover these aspects.

The constructed and validated interventions that were composed of verbs such as “obtain”, “investigate”, “identify” and “establish” are effective for the subset in question. Interventions, in general, were related to the partially compensatory system, in order to encourage a person’s participation in nursing consultation, enabling the identification of risk factors and promoting the construction of knowledge and individuals’ role for autonomy and self-care.

Considering the validated interventions, there was a predominance of the support-education system, which was represented by 299 (51%) of interventions statements, reinforcing PHC nurses’ role in promoting health for the assisted population through health education. However, such educational interventions need to be put into practice. A study with health professionals from southern Brazil reveals that health education for people with diabetes in PHC must be rescued and valued as a working tool based on a dialogic and emancipatory model that guides clinical practices^(26)^.

Therefore, it is necessary for nurses to combine, in their care plan, technologies known as contemporary in care management to achieve satisfactory results, taking into account changes in behavior and lifestyle. Motivational interviewing and the operative group can be cited as methods of monitoring the chronic and peculiar conditions of each person, thus contributing to safe and qualified care^(27)^. These strategies can be included in the indications of diagnoses and nursing interventions.

An important result found was the number of statements of diagnoses allocated in the self-care requirements related to the development. In the development process, a person with diabetic foot ulcer may experience coping difficulties and emotional changes resulting from the condition of life, requiring attention from professionals for these difficulties. Therefore, the survey of these statements also needs to be considered for comprehensive care, suggesting the indication of nursing interventions that promote the need for a multidisciplinary approach^(28)^.

As seen, Orem’s theoretical conception subsidized the proposed subset in a disciplinary and professional knowledge. It is noted that the proposed statements addressed the range of care demands as well as nursing actions, and allowed, through the classification of nursing diagnoses, by self-care requirements, and interventions, by nursing systems, the practical application of this theory in the context of care for the studied patients, which can enhance the relevance of the nursing process, allowing visibility and appreciation.

Orem’s conception aims to identify the reasons that contribute to a person needing care, determining self-care requirements, competence for self-care practice, therapeutic demand and care planning through nursing systems. In this way, it is necessary that the construction of self-care goes through education and reaches physical, psychological and social autonomy^(29)^. Therefore, it is necessary for nurses to know the steps of their assistance again based on data collection, diagnosis and nursing planning, implementation and assessment, considering that the absence of a nursing process can result in care without quality and effectiveness.

Thus, it is printed that the subset on screen is a technical product for documenting a specialized nursing terminology, which can be consulted and used by nurses, which will allow strengthening the standardization of a language specific to the area. Thus, PHC nurses, working in a territory close to patients, will be able to evidence and judge real health needs, which will be fundamental for self-care promotion.

### Study limitations

The study presents as a limitation the fact that the terms have been explored in the literature of the area and focus specifically on people with diabetic foot ulcers, which may not reveal the complexity of care for people with other complications caused by diabetes.

### Contributions to nursing and public health

The subset on screen allows cooperation for nursing practice in the most distant and difficult accesses for people with diabetes, contributing to a systematized care that meets individuals’ peculiarities. Furthermore, as nurses work based on a terminology and a theoretical conception, care becomes targeted, creative, safe and scientific.

## CONCLUSIONS

The objective of this study was achieved from the elaboration of an ICNP^®^ terminological subset for people with diabetic foot ulcers. The 81 statements of diagnoses/outcomes and 583 elaborated nursing interventions are relevant to the literature and converge with PHC nurses’ experience. Thus, this subset shows real health needs, which include the biopsychosocial and spiritual aspects, where nurses can use their clinical reasoning to build and confirm these statements in order to guarantee access, coordination, comprehensiveness and longitudinality, which will be fundamental for promoting self-care aimed at quality of life and recovery.
